# Dinámica familiar y extrafamiliar en adultos mayores con baja adherencia al tratamiento farmacológico en un programa de falla cardiaca en Santander, Colombia

**DOI:** 10.1016/j.aprim.2026.103552

**Published:** 2026-06-16

**Authors:** Constanza Carolina Torres Montenegro, Harold Torres Pinzón, Juan Sebastián Therán León, Luis Eduardo Echeverría Correa

**Affiliations:** a Programa de Especialización en Medicina Familiar, Facultad de Ciencias Médicas y de la Salud, Universidad de Santander (UDES), Bucaramanga, Colombia; b Grupo de Investigación Salud-ComunidUDES SCU, Universidad de Santander (UDES), Bucaramanga, Colombia; c Facultad de Ciencias Médicas y de la Salud, Universidad de Santander (UDES), Bucaramanga, Colombia; d Programa de Falla Cardiaca, Fundación Cardiovascular de Colombia (FCV), Floridablanca, Santander, Colombia

El fallo cardiaco (FC) afecta a más de seis millones de personas en EE. UU. En Colombia, presenta una mortalidad cercana a 6,4 fallecimientos por 100.000 habitantes^1^. La adherencia al esquema neurohormonal —inhibidores del sistema renina-angiotensina, betabloqueadores, antagonistas de mineralocorticoides e inhibidores SGLT2— condiciona descompensaciones, reingresos y mortalidad^2^. En adultos mayores, el funcionamiento familiar y las redes extrafamiliares actúan como determinantes sociales del autocuidado, aunque su caracterización en programas latinoamericanos especializados es escasa^3^. El objetivo del presente trabajo es describir la dinámica familiar y extrafamiliar de adultos mayores con baja adherencia al tratamiento farmacológico inscritos en un programa de insuficiencia cardiaca.

Se realizó un estudio observacional descriptivo de corte transversal (STROBE), entre noviembre de 2025 y febrero de 2026, en un programa de FC de una institución de alta complejidad de Santander, Colombia. Se incluyeron adultos ≥ 60 años con FC y baja adherencia por test de Morisky-Green, capacidad cognitiva conservada y consentimiento informado verbal, se excluyeron pacientes con deterioro cognitivo severo sin cuidador, comorbilidades terminales u hospitalización prolongada. El tamaño muestral, calculado para una proporción esperada de baja adherencia del 9,4%^4^, fue de 125 participantes (264 elegibles; 139 no incluidos por no localización, fallecimiento, hospitalización, cambio de aseguradora o rechazo). Se aplicaron el APGAR familiar (función normal 17–20; disfunción leve 13–16, moderada 10–12, severa ≤ 9)^5^ y el Ecomapa en ocho dominios^6^. El análisis se realizó con SPSS v.27 mediante frecuencias absolutas y relativas con intervalos de confianza (IC) del 95% (método de Wilson) y mediana con rango intercuartílico (RIC) para variables cuantitativas. El protocolo fue aprobado por los comités de ética institucionales; se siguieron los protocolos sobre publicación de datos de pacientes con anonimización mediante codificación alfanumérica y se respetó la privacidad de los sujetos.

La mediana de edad fue 73 años (RIC 65–80); 68,0% varones; 49,6% estrato bajo; mediana de siete medicamentos (RIC 5–8), 81,6% convivían con otras personas y 88,0% tenían cuidador principal (84,8% familiar). El APGAR mostró función normal en 66,4% (IC 95% 57,8–74,1), disfunción leve 20,8% (IC 95% 14,4–28,9), moderada 8,8% (IC 95% 4,8–15,2) y severa 4,0% (IC 95% 1,7–9,0). La disfunción severa concentró mayor proporción de vida en soledad (40,0%), ausencia de cuidador (40,0%) y polifarmacia (mediana 10; RIC 7–12). El Ecomapa evidenció predominio de relaciones fuertes en salud (82,4%), comunidad (76,0%), religión (72,0%), amigos (68,0%) y familia (67,2%), recreación (65,6%), trabajo (78,4%) y educación (96,0%) concentraron vínculos débiles. Las relaciones conflictivas fueron infrecuentes (familia 7,2%; resto < 1%) (tabla 1).

**Tabla 1:** Características sociodemográficas, dinámica familiar (APGAR) y dinámica extrafamiliar (Ecomapa) de adultos mayores con fallo cardiaco y baja adherencia farmacológica (n = 125)

A. Características sociodemográficas y de cuidado			
Variable	n / mediana	% / RIC	Observación
Edad (años), mediana (RIC)	73	65–80	rango 60–99
Sexo masculino	85	68,0%	—
Estrato socioeconómico bajo	62	49,6%	—
Nivel educativo ≤ básica primaria	76	60,8%	—
Régimen subsidiado	75	60,0%	—
Medicamentos prescritos, mediana (RIC)	7	5–8	rango 2–15
Convive con otras personas	102	81,6%	—
Vive solo	23	18,4%	—
Cuidador principal (familiar)	110 (106)	88,0% (84,8%)	—
B. Funcionamiento familiar — APGAR familiar (rango 0–20)			
Categoría	n	%	IC 95%
Función normal (17–20)	83	66,4%	57,8–74,1
Disfunción leve (13–16)	26	20,8%	14,4–28,9
Disfunción moderada (10–12)	11	8,8%	4,8–15,2
Disfunción severa (≤ 9)	5	4,0%	1,7–9,0
*Subgrupo con disfunción severa (n* *=* *5): vive solo 40,0%; sin cuidador 40,0%; polifarmacia mediana 10 (RIC 7–12); varones 80,0%.*	—	—	—
C. Dinámica extrafamiliar — Ecomapa por dominio (% del total)			
Dominio	Fuerte (%)	Débil (%)	Conflictiva (%)
Salud	82,4	17,6	0,0
Comunidad	76,0	23,2	0,8
Religión	72,0	27,2	0,8
Amigos	68,0	31,2	0,8
Familia	67,2	25,6	7,2
**Recreación**	**33,6**	**65,6**	**0,8**
**Trabajo**	**21,6**	**78,4**	**0,0**
**Educación**	**4,0**	**96,0**	**0,0**

RIC: rango intercuartílico; IC 95%: intervalo de confianza del 95% calculado por método de Wilson.

Las celdas resaltadas en cursiva señalan el subgrupo con disfunción severa (n = 5); las filas en negritas señalan los dominios extrafamiliares con predominio de vínculos débiles. Las proporciones del subgrupo severo deben interpretarse con cautela dada la baja frecuencia absoluta.

El uso conjunto del APGAR familiar y el Ecomapa permitió identificar un subgrupo de especial vulnerabilidad —adultos mayores con FC, vida en soledad, ausencia de cuidador y polifarmacia— susceptible de intervención focalizada desde atención primaria. El predominio de funcionalidad familiar coincide con la literatura latinoamericana en enfermedades crónicas^3^, no obstante, la concentración de disfunción severa con polifarmacia subraya un grupo prioritario habitualmente invisibilizado en programas especializados. La debilidad de vínculos en recreación, trabajo y educación, característica de una vida cotidiana con baja participación social, se ha asociado con peor adherencia y pronóstico^2^.

Las limitaciones incluyen el diseño descriptivo, sin posibilidad de inferencia causal ni análisis estratificado por sexo o género; el autorreporte de adherencia mediante test de Morisky-Green, susceptible de sesgo de deseabilidad social; y la no localización del 38,6% de los elegibles, que pudo sesgar la muestra hacia perfiles clínicamente más estables. La incorporación rutinaria del APGAR familiar y el Ecomapa —instrumentos de bajo coste y alta factibilidad— al seguimiento ambulatorio podría priorizar acompañamiento integrado y reducir reingresos en el subgrupo más vulnerable, articulando el ámbito clínico con los recursos familiares y comunitarios disponibles.

## Sitio del estudio

Programa de Falla Cardiaca de la Fundación Cardiovascular de Colombia (FCV), Floridablanca, Santander, Colombia.

## Financiación

El estudio ha sido financiado con recursos propios de los investigadores. La presente investigación no ha recibido ayudas específicas provenientes de agencias del sector público, sector comercial o entidades sin ánimo de lucro. Estudio financiado con recursos propios de los investigadores.

Los autores declaran no tener ninguna relación financiera ni personal con personas u organizaciones que pudiera haber influido de manera inapropiada en el presente trabajo.

## Consideraciones éticas

El protocolo fue aprobado por el Comité de Bioética de la Universidad de Santander y por el Comité de Ética e Investigación de la Fundación Cardiovascular de Colombia. Clasificado como investigación sin riesgo (Resolución 8430/1993, Ministerio de Salud de Colombia, Artículo 16). Se obtuvo consentimiento informado verbal de todos los participantes con registro auditable.

El protocolo fue aprobado por el Comité de Bioética de la Universidad de Santander (Acta No. [a completar por la autora] de fecha [a completar]) y por el Comité de Ética e Investigación de la Fundación Cardiovascular de Colombia, FCV (Acta No. [a completar] de fecha [a completar]). El estudio se clasificó como investigación sin riesgo conforme a la Resolución 8430 de 1993 del Ministerio de Salud de Colombia y se desarrolló de acuerdo con los principios de la Declaración de Helsinki (revisión 2013), la Ley 23 de 1981 y la Ley 1581 de 2012 de protección de datos personales. Se obtuvo consentimiento informado verbal de todos los participantes conforme al Artículo 16 de la Resolución 8430/1993, con registro auditable de la aceptación. Los datos fueron anonimizados mediante códigos alfanuméricos y almacenados en nube institucional con acceso restringido.

## Declaración de la IA generativa y las tecnologías asistidas por IA en el proceso de escritura

Durante la preparación de esta versión adaptada los autores utilizaron Claude (Anthropic) con el fin de apoyar la edición lingüística y la verificación de coherencia estructural conforme a las normas de la sección Cartas Científicas. Tras utilizar dicha herramienta, los autores revisaron y editaron el contenido según necesidad, asumiendo la plena responsabilidad del contenido de la publicación.

## Contribución de autoría (taxonomía CRediT)

CCTM: Conceptualización, investigación, recolección de datos, redacción del borrador original, administración del proyecto. HTP: Metodología, análisis formal, supervisión, revisión y edición crítica del manuscrito. JSTL: Revisión metodológica crítica, análisis formal, redacción — revisión y edición, validación científica. LEEC: Conceptualización, acceso a la población de estudio, supervisión clínica, validación, revisión crítica del manuscrito.

Todos los autores han leído y aprobado la versión reformulada del manuscrito y asumen la responsabilidad del contenido publicado conforme a los criterios del ICMJE.

## Conflicto de intereses

Los autores declaran ausencia de conflicto de intereses.
